# The management of light chain (AL) amyloidosis in Europe: clinical characteristics, treatment patterns, and efficacy outcomes between 2004 and 2018

**DOI:** 10.1038/s41408-023-00789-8

**Published:** 2023-01-25

**Authors:** Giovanni Palladini, Stefan Schönland, Giampaolo Merlini, Paolo Milani, Arnaud Jaccard, Frank Bridoux, Meletios A. Dimopoulos, Sriram Ravichandran, Ute Hegenbart, Wilfried Roeloffzen, M. Teresa Cibeira, Hermine Agis, Monique C. Minnema, Rui Bergantim, Roman Hájek, Cristina João, Alexandros Leonidakis, Giorgos Cheliotis, Pieter Sonneveld, Efstathios Kastritis, Ashutosh Wechalekar

**Affiliations:** 1 Amyloidosis Research and Treatment Center, Foundation “Instituto di Ricovero e Cura a Carattere Scientifico (IRCCS) Policlinico San Matteo”, Department of Molecular Medicine, University of Pavia, Pavia, Italy; 2 Medical Department V, Amyloidosis Center Heidelberg, University of Heidelberg, Heidelberg, Germany; 3 National Amyloidosis Center and Hematology Unit, CHU Limoges, Limoges, France; 4 Nephrology Unit, CHU Poitiers, Poitiers, France; 5 Department of Clinical Therapeutics, National and Kapodistrian University of Athens, School of Medicine, Athens, Greece; 6 National Amyloidosis Centre, University College London, London, UK; 7 Amyloidosis Centre of Expertise Department of Internal Medicine, Faculty of Medical Sciences, University Medical Center Groningen, Groningen, Netherlands; 8 Amyloidosis and Myeloma Unit, Department of Hematology, Hospital Clinic de Barcelona, IDIBAPS, Barcelona, Spain; 9 Department of Internal Medicine I, Division of Oncology, Medical University Vienna, Vienna, Austria; 10 Department of Hematology, University Medical Center Utrecht, Utrecht, Netherlands; 11 Clinical Hematology, Centro Hospitalar São João, Porto, Portugal; i3S - Instituto de Investigação e Inovação em Saúde, University of Porto, Porto, Portugal; Cancer Drug Resistance Group, IPATIMUP - Institute of Molecular Pathology and Immunology of the University of Porto, Porto, Portugal; Clinical Hematology, FMUP - Faculty of Medicine, University of Porto, Porto, Portugal; 12 Department of Haematooncology, University Hospital Ostrava, and Department of Haematooncology, Faculty of Medicine, University of Ostrava, Ostrava, Czech Republic; 13 Hematology Department, Champalimaud Center for the Unknown, Lisbon, Portugal; 14 Health Data Specialists, Dublin, Ireland; 15 Erasmus MC Cancer Institute, Rotterdam, Netherlands

**Keywords:** Haematological cancer, Haematological cancer

## Abstract

Systemic light-chain (AL) amyloidosis is a rare and debilitating disease. Advances have been made in new treatments in recent years, yet real-world data on the management of the disease are scarce. EMN23 is a retrospective, observational study of patients who initiated first-line treatment in 2004–2018 in Europe, presenting the demographics, clinical characteristics, treatment patterns, and outcomes, from 4480 patients. Regimens based on bortezomib were the most frequently used as first-line therapy; only 6.2% of the patients received autologous stem cell transplant. Hematologic responses improved post-2010 (67.1% vs 55.6% pre-2010). The median overall survival (OS) was 48.8 (45.2–51.7) months; 51.4 (47.3–57.7) months pre-2010 and 46.7 (41.3–52.2) months post-2010. Early mortality was 13.4% and did not improve (11.4% vs 14.4% pre- and post-2010); furthermore, it remained high in patients with advanced cardiac disease (over 39% for stage IIIb). There was a significant improvement for stage IIIa (14.2 vs 30.7 months, *p* = 0.0170) but no improvement for stage IIIb patients (5.0 vs 4.5 months). This European real-world study of AL-amyloidosis emphasizes the unmet needs of early diagnosis, and the lack of improvement in survival outcomes of the frail stage IIIb population, despite the introduction of new therapies in recent years.

## Introduction

Systemic light chain (AL) amyloidosis is a rare disease caused by misfolded clonal immunoglobulins produced by a plasma cell or, less often, a B cell clone [1, 2]. The immunoglobulin oligomers are toxic to tissues and organs [3], and amyloid deposition disrupts their normal function [4], leading to debilitating and commonly lethal complications [1, 2]. AL amyloidosis is associated with non-specific symptoms and substantial heterogeneity in clinical presentation [1, 2]. Correct diagnosis requires a high degree of suspicion and specialized techniques [5, 6], while the multisystemic nature of this disease requires multidisciplinary management and significant healthcare resources use [1, 2]. As a result, diagnosis and treatment of AL amyloidosis is often delayed [7] until after irreversible organ damage has occurred.

Current treatments for AL amyloidosis target the plasma/B cell clone aiming to reduce the production of toxic light chains. Until recently there were no approved therapies for AL amyloidosis and this disease was managed with adapted off-label therapies indicated for multiple myeloma or Waldenström’s Macroglobulinemia [8] following advances in these areas. New treatments for the underlying clonal disease in the past 10–15 years (such as bortezomib) [9–17] changed the therapeutic management of AL amyloidosis, but treatment patterns remained heterogeneous.

Because of the above, the approach to the diagnosis and management of AL amyloidosis has been based mostly on single referral center studies and much less on prospective or randomized studies [15, 17–19]. Data from tertiary referral centers from US indicate improvements over the past decades [20, 21] but a comprehensive, real-world overview is lacking. This is especially relevant for Europe, which is characterized by variations in the organization of the health care systems and allocation of healthcare resources in the different countries, including availability of diagnostic methods and approved therapies.

## Methods

### Study design

EMN23 is a retrospective, observational study aiming to collect real-world evidence for the management of systemic AL amyloidosis in patients who initiated first-line treatment from January 2004 to December 2018 in Europe. The study was sponsored by the European Myeloma Network (EMN). The duration from initiation until the database lock was approximately 24 months (Q2-2019 to Q2-2021). Collection of data and analysis of the main outcomes of interest consisted of patient demographic and clinical characteristics at diagnosis, treatments (at first and second line), and survival outcomes (overall survival (OS); time on treatment (ToT)) analyzed by treatment initiation period, cardiac stage, and hematologic responses. Landmark analyses on time to event endpoints for hematologic responses at 3 and 6 months, were performed for patients with available data.

One of the study aims was to investigate potential differences between the cohorts of patients who initiated first-line treatment in 2004–2010 (pre-2010) and 2011–2018 (post-2010), in order to depict changes in disease management secondary to the wider introduction of bortezomib-based regimens after 2010, especially of bortezomib, cyclophosphamide, and dexamethasone (VCD), in the everyday clinical practice [11, 12, 14, 22].

Patient data were collected from ten European countries: Austria, Czech Republic, France, Germany, Greece, Italy, the Netherlands, Portugal, Spain, and the United Kingdom (UK). According to local regulations, data were retrieved from patients’ medical records (paper/electronic and electronic databases) and were transferred directly to the study database (Germany, Greece, Italy, Spain, and the UK) and/or via an electronic case report form (all countries except Italy, Spain, and the UK).

The hematologic responses were assessed by investigators according to the protocol as per consensus criteria. In best hematologic responses, and in 3- and 6-month landmark analyses, patients who died less than 3 months after first-line treatment initiation and did not have a post-baseline assessment, and who were to be included in the landmark analysis were categorized under the “no-response” group. Patients who participated in clinical trials or patients who were under the “no-response” group were not included in the estimation of the hematologic response rates. Organ involvement was assessed according to criteria defined in each chronological period [23, 24].

The study protocol and amendments were reviewed by an Independent Ethics Committee or Institutional Review Board as appropriate per local laws and the study was conducted in accordance with Good Pharmacoepidemiology Practice (GPP) guidelines. Data collection and source data verification were always performed in accordance with local requirements and legislations.

### Statistical analysis

Descriptive statistics for continuous variables included mean, standard deviation, median, Q1, Q3 quartile, minimum value, maximum value and 95% CI. Categorical variables were described using absolute frequencies and proportions. Significant differences between subgroups were examined using Mann-Whitney non-parametric test continuous variables and chi-square tests for categorical variables. Descriptive statistics for time-to-event variables (OS, ToT) were estimated using the Kaplan–Meier method, while to compare the survival distributions between subgroups, log rank test was used, based on the nature of the data.

Overall Response Rate (ORR) was defined as the proportion of patients with a best hematologic response of partial response (PR) or better. Patients without available response data were excluded from the denominator.

OS was defined as the time from treatment initiation (first treatment dose; the date of ASCT was used for the patients who underwent upfront ASCT) to death due to any cause or to the last follow-up (censored).

ToT was defined as the time from treatment initiation to discontinuation of first line treatment for any reason (e.g., scheduled treatment completion, disease progression (PD), toxicity, death, lost to follow-up). Patients who did not discontinue treatment were censored at the date of the last follow-up or on 31 December 2018 (whichever occurred first).

Although no formal hypothesis testing was performed, comparisons between groups were statistically assessed to provide a clearer evaluation of the results. All reported *p* values were two-sided, and *p* < 0.05 was considered statistically significant. All analyses were performed using SAS (v9.4 SAS Institute, Cary, NC). This study is registered with ClinicalTrials.gov, NCT04937777.

## Results

Overall, 4480 patients were enrolled; Table S1 lists the participating centers. Patients who participated in clinical trials (*N* = 177 for first-line treatment and *N* = 38 for second line) were only included in the analyses for patient characteristics. Table 1 presents the demographic and clinical characteristics at diagnosis for patients who started first-line treatment in 2004–2018 (*N* = 4480), pre-2010 (*N* = 1415), and post-2010 (*N* = 3065).

**Table 1:** Patient demographic and disease characteristics at diagnosis, overall (2004–2018), and by first-line treatment initiation period (pre- and post-2010).

Period^a^	2004–2018	Pre-2010	Post-2010
*Patients, N*	4480	1415	3065
*Gender, N (%)*^b^			
Male	2615 (58.4)	819 (57.9)	1796 (58.6)
Female	1865 (41.6)	596 (42.1)	1269 (41.4)
Male/female ratio	1.4/1	1.4/1	1.4/1
*Age, years*			
Patients, *N*	4479	1415	3064
Mean (SD)	64.4 (10.2)	63.6 (10.4)	64.8 (10.1)
Median	65.0	64.0	66.0
Min–Max	29.0–91.0	30.0–89.0	29.0–91.0
Q1–Q3	57.0–72.0	56.0–71.0	58.0–72.0
*Age groups, N (%)*^b^			
Reported	4479	1415	3064
<60 years	1386 (30.9)	484 (34.2)	902 (29.4)
60–64 years	704 (15.7)	227 (16.0)	477 (15.6)
65–74 years	1661 (37.1)	494 (34.9)	1167 (38.1)
≥75 years	728 (16.3)	210 (14.8)	518 (16.9)
Not reported	1 (0.0)	–	1 (0.0)
*Time from first symptoms to diagnosis, months*			
Patients, *N*	967	258	709
Mean (SD)	11.7 (22.4)	12.0 (23.2)	11.6 (22.1)
Median	5.1	5.4	5.0
Min–Max	0.0–244.9	0.0–244.9	0.0–236.5
Q1–Q3	1.3–12.4	1.1–13.0	1.4–12.0
*Time from diagnosis to first–line treatment, months*			
Patients, *N*	4360	1401	2959
Mean (SD)	1.5 (3.8)	1.5 (2.6)	1.5 (4.2)
Median	0.7	0.7	0.8
Min–Max	0.0–80.8	0.0–34.0	0.0–80.8
Q1–Q3	0.5–1.4	0.5–1.5	0.5–1.4
*Mayo2004/European cardiac stage, N (%)*^b^			
Reported	3775 (84.3)	859 (60.7)	2916 (95.1)
I	696 (18.4)	184 (21.4)	512 (17.6)
II	1413 (37.4)	347 (40.4)	1066 (36.6)
IIIa	1003 (26.6)	150 (17.5)	853 (29.3)
IIIb	663 (17.6)	178 (20.7)	485 (16.6)
Not reported	705 (15.7)	556 (39.3)	149 (4.9)
*Revised stage (Mayo2012), N (%)*^b^			
Reported	3065 (68.4)	652 (46.1)	2413 (78.7)
I	608 (19.8)	157 (24.1)	451 (18.7)
II	743 (24.2)	163 (25.0)	580 (24.0)
III	896 (29.2)	157 (24.1)	739 (30.6)
IV	818 (26.7)	175 (26.8)	643 (26.6)
Not reported	1415 (31.6)	763 (53.9)	652 (21.3)
*Organ involvement, N (%)*^b,c^			
Heart	3041 (67.9)	906 (64.0)	2135 (69.7)
Kidneys	2961 (66.1)	937 (66.2)	2024 (66.0)
Soft tissue	802 (17.9)	193 (13.6)	609 (19.9)
Nervous system	646 (14.4)	199 (14.1)	447 (14.6)
Liver	640 (14.3)	231 (16.3)	409 (13.3)
Gastrointestinal tract	283 (6.3)	68 (4.8)	215 (7.0)
Lung	39 (0.9)	13 (0.9)	26 (0.9)
*Number of organs involved, N (%)*^b^			
Reported	4329 (96.6)	1282 (90.6)	3047 (99.4)
1	1536 (35.5)	413 (32.2)	1123 (36.9)
2	1784 (41.2)	560 (43.7)	1224 (40.2)
≥3	1009 (23.3)	309 (24.1)	700 (23.0)
Not reported	151 (3.4)	133 (9.4)	18 (0.6)
*ECOG PS, N (%)*^b^			
Reported	3432 (76.6)	728 (51.4)	2704 (88.2)
0	623 (18.2)	70 (9.6)	553 (20.5)
1	1411 (41.1)	289 (39.7)	1122 (41.5)
2	1010 (29.4)	221 (30.4)	789 (29.2)
3	362 (10.5)	131 (18.0)	231 (8.5)
4	26 (0.8)	17 (2.3)	9 (0.3)
Not reported	1048 (23.4)	687 (48.6)	361 (11.8)

*ECOG PS* Eastern Cooperative Oncology Group performance status, *Max* maximum value, *Min* minimum value, *N* number of patients, *Q1* first quartile, *Q3* third quartile, *SD* standard deviation.

^a^Pre- and post-2010 indicate the periods of 2004–2010 and 2011–2018, respectively.

^b^Percentages are calculated using the total number of patients with available data in each column as the denominator.

^c^Patients could have >1 involved organs.

In 967 (21.6%) patients with available data, the median time from symptoms to diagnosis was 5.1 months (5.4 and 5.0 months pre- and post-2010, respectively) and the median time from diagnosis to initiation of first-line treatment was 0.7 months.

Clonal disease was immunoglobulin light chain only in 41.0% while 4.4% of patients had IgM-related AL amyloidosis (Table S2). Heart (67.9%) and kidneys (66.1%) were the most frequently involved organs. Most patients (74.1%) had ≤2 organs involved and 34.3% had only one organ involved.

Cardiac stage (Mayo 2004 with European modification) [25, 26] at the time of diagnosis was available for 3775 (84.3%) patients, of whom 696 (18.4%) were stage I, 1413 (37.4%) stage II, 1003 (26.6%) stage IIIa, and 663 (17.6%) stage IIIb. Per Mayo 2012 staging [27] (available for 3065 patients), 19.8%, 24.2%, 29.2%, and 26.7% were rated as stage I, II, III, and IV, respectively. Cardiac stage distribution at diagnosis was similar for the two chronological periods (Table 1).

### First-line treatment

Table 2 depicts regimen categories and the most common individual regimens that were used as first-line therapies. Bortezomib-based regimens (54.8%, *N* = 2454) were the most common first-line therapy, followed by chemotherapy (19.4%, *N* = 869) [mostly melphalan with dexamethasone (MDex)], and immunomodulatory drug (IMiD)-based regimens *(N* = 488, 10.9%). High-dose melphalan (HDM) with ASCT was used at first line in 6.2% (*N* = 278); 74.1% (*N* = 206) received induction prior to HDM-ASCT (bortezomib-based in 68.0% and IMiDs in 18.0%). First-line treatments among participating countries are shown in Table S3.

**Table 2:** First-line treatment regimens, overall (2004–2018), and by first-line treatment initiation period (pre- and post-2010).

Period^a^	2004–2018	Pre-2010	Post-2010
Regimen groups, *N* (%)^b^			
Patients, *N*	4480	**1415**	**3065**
Bor-based	2454 (54.8)	163 (11.5)	2291 (74.7)
IMiD-based	488 (10.9)	429 (30.3)	59 (1.9)
Chemo	869 (19.4)	603 (42.6)	266 (8.7)
Rituximab-based	95 (2.1)	29 (2.0)	66 (2.2)
Dara-based	21 (0.5)	–	21 (0.7)
Steroids	49 (1.1)	38 (2.7)	11 (0.4)
ASCT	278 (6.2)	108 (7.6)	170 (5.5)
Clinical trial	177 (4.0)	35 (2.5)	142 (4.6)
Other regimen groups	49 (1.1)	10 (0.7)	39 (1.3)
Individual regimens, *N* (%)			
Patients, *N*	4480	1415	3065
ASCT	278 (6.2)	108 (7.6)	170 (5.5)
VCD	653 (14.6)	23 (1.6)	630 (20.6)
MDex	707 (15.8)	458 (32.4)	249 (8.1)
VMD	210 (4.7)	44 (3.1)	166 (5.4)
RD	25 (0.6)	8 (0.6)	17 (0.6)
VD	401 (9.0)	79 (5.6)	322 (10.5)
CTD	357 (8.0)	337 (23.8)	20 (0.7)
Clinical trial	177 (4.0)	35 (2.5)	142 (4.6)
Other individual regimens	1672 (37.3)	323 (22.8)	1349 (44.0)

*ASCT* autologous stem cell transplantation, *Bor* bortezomib, *Chemo* chemotherapy, *CTD* cyclophosphamide, thalidomide, and dexamethasone, *Dara* daratumumab, *IMiD* immunomodulatory drugs, *MDex* melphalan and dexamethasone, *N* number of patients, *RD* lenalidomide and dexamethasone, *VCD* bortezomib, cyclophosphamide, and dexamethasone, *VD* bortezomib and dexamethasone, *VMD* bortezomib, melphalan, and dexamethasone.

^a^Pre- and post-2010 indicate the periods of 2004–2010 and 2011–2018, respectively.

^b^Percentages are calculated using the total number of patients in each column as the denominator.

Bold values indicate the number of patient subgroups pre-2010 and post-2010.

There was a significant change of first-line treatment type in the two chronological periods. Pre-2010, most patients received chemotherapy (in 42.6%, MDex in 32.4%), followed by IMiD-based regimens (30.3%), and bortezomib-based therapy (in 11.5%) while, after 2010, bortezomib-based regimens were used in the majority of patients (74.7%); VCD was the most frequently used bortezomib-based regimen (20.6% of all regimens post-2010), followed by bortezomib and dexamethasone (VD, 10.5%) and bortezomib with MDex (VMD, 5.4%). ASCT was performed in 7.6% of patients pre-2010 and in 5.5% post-2010.

Pre-2010, chemotherapy (mostly MDex) was the most frequently used regimen across all cardiac stages (range: 39.7% for stage I to 50.7% for stage IIIa). Post-2010, bortezomib-based regimens, and VCD in particular, were used in the majority of patients across all cardiac stages (range: 64.8% for stage I to 82.3% for stage IIIb) (Table S4). In patients with kidney involvement bortezomib-based therapy was used in 54.9%, chemotherapy in 19.2%, and IMiDs in 10.4%. Among patients with nerve involvement, 44.0% received bortezomib-based therapy, whereas 31.9% and 7.4% were given chemotherapy and IMiDs, respectively (Table S5). Most patients with IgM-related amyloidosis received rituximab-based regimens at first line, 33.3% (*N* = 23) and 39.8% (*N* = 51), pre- and post-2010, respectively (Table S6).

### Response to first-line treatment

After first-line therapy, 63.4% of patients with available information achieved at least hematologic PR (including CR: 23.2% and VGPR: 21.2%, Table S7). The hematologic response rate was 68.4% with bortezomib-based regimens (CR: 26.1%, VGPR: 25.7%); 49.0% (CR: 14.3%, VGPR: 13.4%) with chemotherapy, and 53.7% (CR: 17.5%, VGPR: 9.3%) with IMiD-based regimens (Table S8). Hematologic response rates were similar among bortezomib-based regimens: 63.7% (CR: 19.2%, VGPR: 27.7%) for VCD, 62.1% (CR: 12.7%, VGPR: 27.4%) for VD, and 57.3% (CR: 20.5%, VGPR: 25.1%) for VMD. In ASCT-treated patients, 88.0% achieved ≥PR (CR: 43.4%, VGPR: 27.0%) (Table S9). The proportion of patients who failed to achieve a response or died within 3 months after starting therapy without any response assessment, pre- and post-2010, was 35.6% and 31.3% with bortezomib-based, 50.5% and 52.6% with chemotherapy, 43.9% and 65.4% with IMiD-based regimens, and 24.8% and 3.7% with ASCT (Table S10).

Hematologic response rates significantly improved post-2010 (67.1% vs 55.6% pre-2010, *p* < 0.001), with deeper responses (25.0% vs 19.5% CRs and 25 4% vs 12.4% VGPRs). As a consequence of the improvement in hematologic responses non-responding patients significantly decreased from 44.4% (SD: 16.7%, PD: 19.6%) pre-2010 to 32.9% (SD: 14.8%, PD: 9.4%) post-2010 (Table S11).

Among patients with available information, hematologic response was achieved in 58.6% of patients at 3 months from the start of first-line therapy (1594/2720) and in 70.1% of patients at 6 months (1633/2329). Three-month ORR was 45.6% (CR + VGPR: 24.8%) pre-2010 and 63.6% (CR + VGPR: 38.9) post-2010, and 6-month ORR was 58.3% (CR + VGPR: 31.0%) and 75.1% (CR + VGPR: 54.5%), respectively (Table S12).

Hematologic response rates at 3 months were 64.1% for bortezomib-based regimens, 42.2% for chemotherapy and 42.8% for IMiD-based regimens. At the 6-month landmark, response rates for patients with available information were 74.5% for bortezomib-based regimens, 54.1% for chemotherapy, and 55.1% for IMiD-based regimens (Table S13).

Median ToT for first-line therapy was 4.7 (range: 4.4–5.0) months; similar in the pre- and post-2010 era (4.7 vs 4.8 months) and was shorter in patients with more advanced disease stages (Table S14).

### Second-line therapy

Overall, 1759 patients received second-line treatment. After the second-line therapy, 58.7% of patients with available information achieved at least hematologic PR (including CR: 15.3% and VGPR: 20.1%, Table S7). Median time from start of first line to second-line therapy was 9.5 (0.6–165.7) months and was shorter post-2010 (8.6 vs 11.3 months). Pre-2010, bortezomib-based regimens were the most frequent second-line treatment (*N* = 349, 45.0%) while post-2010 IMiD-based regimens became the major choice (*N* = 410, 41.7%) (Table 3). Notably, 15.6% of patients who received bortezomib at first line and required a second-line treatment, received bortezomib-based therapy again (Table S15). Per cardiac stage, 50.8% of patients with stage I, 46.6% with stage II, 33.1% with stage IIIa, and only 18.6% with stage IIIb received second-line treatment. Among patients who received first-line ASCT and required second-line therapy (*N* = 122), 30.3% received bortezomib-based, and 33.6% IMiD-based regimens. Salvage therapy for patients who failed to respond to primary therapy was based on bortezomib and IMiDs in most cases, with bortezomib used in most patients after primary failure to IMIDs (55.7%), and IMiDs (51.8%) in most patients after primary failure to bortezomib.

**Table 3:** Second-line treatment regimens, overall (2004–2018), and by first-line treatment initiation period (pre- and post-2010).

Period^a^	2004–2018	Pre-2010	Post-2010
Regimen groups, *N* (%)^b^			
Patients, *N*	1759	775	984
Bor-based	548 (31.2)	349 (45.0)	199 (20.2)
IMiD-based	639 (36.3)	229 (29.5)	410 (41.7)
Chemo	243 (13.8)	124 (16.0)	119 (12.1)
Rituximab-based	57 (3.2)	11 (1.4)	46 (4.7)
Dara-based	54 (3.1)	–	54 (5.5)
Steroids	9 (0.5)	8 (1.0)	1 (0.1)
ASCT	128 (7.3)	32 (4.1)	96 (9.8)
Clinical trial	38 (2.2)	12 (1.5)	26 (2.6)
Other regimen groups	43 (2.4)	10 (1.3)	33 (3.4)
Individual regimens, *N* (%)			
Patients, *N*	1759	775	984
ASCT	128 (7.3)	32 (4.1)	96 (9.8)
VCD	156 (8.9)	95 (12.3)	61 (6.2)
MDex	138 (7.8)	71 (9.2)	67 (6.8)
VMD	31 (1.8)	11 (1.4)	20 (2)
RD	382 (21.7)	53 (6.8)	329 (33.4)
VD	270 (15.3)	203 (26.2)	67 (6.8)
CTD	119 (6.8)	80 (10.3)	39 (4)
Clinical trial	38 (2.2)	12 (1.5)	26 (2.6)
Other individual regimens	497 (28.3)	218 (28.1)	279 (28.4)

*ASCT* autologous stem cell transplantation, *Bor* bortezomib, *Chemo* chemotherapy, *CTD* cyclophosphamide, thalidomide, and dexamethasone, *Dara* daratumumab, *IMiD* immunomodulatory drugs, *MDex* melphalan and dexamethasone, *N* number of patients, *RD* lenalidomide and dexamethasone, *VCD* bortezomib, cyclophosphamide, and dexamethasone, *VD* bortezomib and dexamethasone, *VMD* bortezomib, melphalan, and dexamethasone.

^a^Pre- and post-2010 indicate the periods of 2004–2010 and 2011–2018, respectively.

^b^Percentages are calculated using the total number of patients in each column as the denominator.

### Survival

The median follow-up for the whole cohort was 54.5 (51.4–57.8) months (Table S16). The median OS was 48.8 (45.2–51.7) months; 51.4 (47.3–57.7) months for the pre-2010 group and 46.7 (41.3–52.2) months for the post-2010 group (Table S17).

**Fig. 1:**
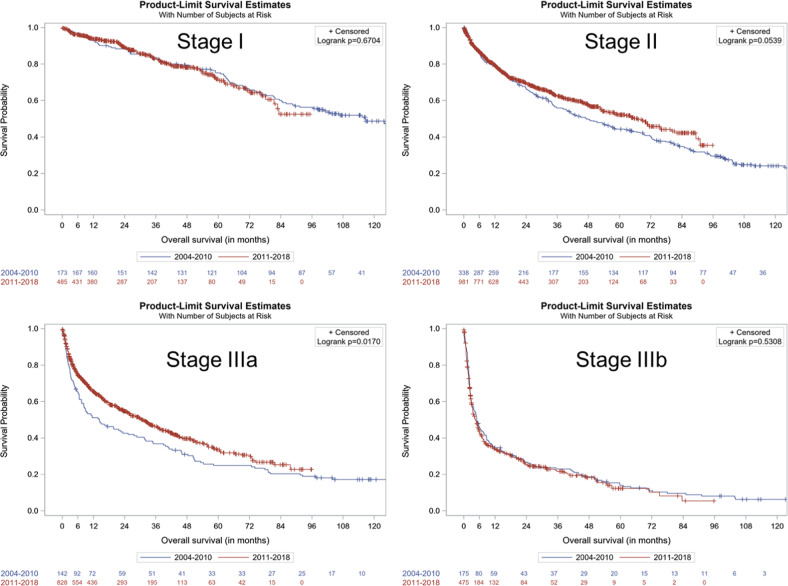
Kaplan–Meier graph of overall survival by first-line treatment initiation period (pre- and post-2010) and Mayo2004/European stage at diagnosis. Stage I, Stage II, Stage IIIa and Stage IIIb represent the comparison of survival curves in the pre- and post-2010, separately for the respective patient subgroups defined by Mayo 2004/European stage at diagnosis.

Early mortality (i.e., within 3 months from start of therapy) was 13.4%, did not improve (11.4% in the pre-2010 and 14.4% in the post-2010 era, Table S18) and was higher in patients with severe cardiac disease in both pre-2010 and post-2010 periods: 1.1% and 2.1% for stage I patients, 7.8% and 7.7% for stage II, 22.7% and 15.6% for stage IIIa, and 39.3% and 39.8% for stage IIIb, respectively (Table 4). The median OS for stage I patients was 116.5 months for the pre-2010 group and was not reached for post-2010 patients (log-rank *p* = 0.670); for stage II patients, median OS was 47.3 months pre-2010 and 67.0 months post-2010 (log-rank *p* = 0.0539); for stage IIIa patients was 14.2 and 30.7 months, respectively (log-rank *p* = 0.0170) and for stage IIIb patients, 5.0 and 4.5 months (log-rank *p* = 0.530), respectively (Fig. 1). The OS per revised Mayo 2012 was not reached in stage I patients, was 69.7 in stage II patients, 24.7 in stage III patients, and 10.6 months for stage IV patients (Fig. S1).

**Table 4:** Early mortality rates by cardiac stage and first-line treatment initiation period.

Response		Stage I		Stage II		Stage IIIa		Stage IIIb		Unknown	
		Pre-2010	Post-2010	Pre-2010	Post-2010	Pre-2010	Post-2010	Pre-2010	Post-2010	Pre-2010	Post-2010
Patients	*N* (%)	184	512	347	1,066	150	853	178	485	556	149
Death prior to 3 months from first-line initiation	Yes	2 (1.1)	11 (2.1)	27 (7.8)	82 (7.7)	34 (22.7)	133 (15.6)	70 (39.3)	193 (39.8)	28 (5.0)	22 (14.8)
	No	171 (92.9)	478 (93.4)	311 (89.6)	902 (84.6)	108 (72.0)	696 (81.6)	105 (59.0)	283 (58.4)	524 (94.2)	123 (82.6)
	Clinical trial	11 (6.0)	23 (4.5)	9 (2.6)	82 (7.7)	8 (5.3)	24 (2.8)	3 (1.7)	9 (1.9)	4 (0.7)	4 (2.7)

The OS was similar between regimen groups (45.3 vs 48.6 vs 41.1 months for bortezomib-, IMiD-, and chemotherapy-based therapy, respectively), while the 3-month OS rate of patients undergoing ASCT was 97.8%. Median OS for patients with stage I disease was not reached for bortezomib-based regimens and was 81.1 and 88.5 months for IMiD-based and chemotherapy regimens, respectively. For stage II, the median OS was 67.0 vs 39.4 vs 44.8 and for stage IIIa 32.8 vs 7.3 vs 14.3 for bortezomib-based, IMiD-based, and chemotherapy regimens, respectively. However, in patients with stage IIIb disease median OS was poor across all regimen groups (4.6 vs 5.3 vs 4.6 respectively) (Table S19). The median OS in patients with IgM-related AL amyloidosis was 39.7 months (51.7 months pre-2010 and 29.8 months post 2010 era, *p* = 0.2772; Table S20).

**Fig. 2:**
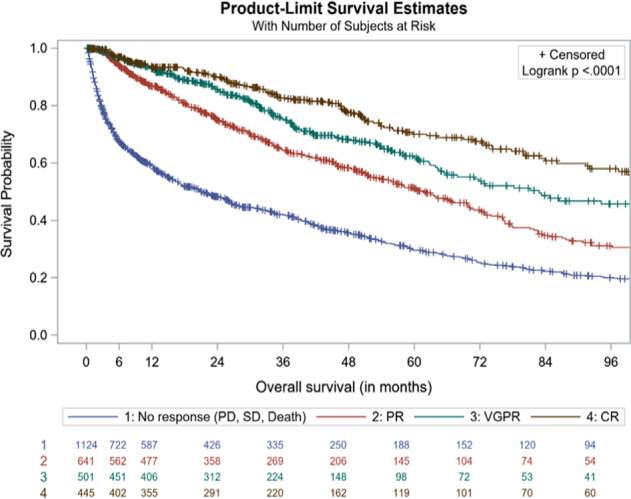
Kaplan–Meier graph of OS by hematologic response at 3 months after first-line treatment initiation, 2004–2018. CR complete response, PD progressive disease, PFS progression-free survival, PR partial response, SD stable disease, VGPR very good partial response.

To evaluate the effect of early hematologic responses on OS we performed a landmark analysis at 3 and at 6 months after treatment initiation. In 2711 evaluable patients the median OS for patients achieving CR at 3 months was 109.4 (range: 97.3–149.7) months, 82.8 (range: 66.0–103.7) months with VGPR, 61.7 months with PR (range: 52.7–69.8), and 21.0 (range: 16.8–25.9) months for those who did not achieve a response (*p* < 0.0001 by log-rank; Fig. 2). At 6 months, the median OS for patients (*N* = 2322) with CR was 142.6 (range: 104.2−NR) months vs 78.2 months for VGPR (range: 64.5–100.8), 59.9 (range: 51.8–69.8) months for PR and 20.2 (range: 14.8–24.3) months for those who did not achieve a response (*p* < 0.0001 by log-rank; Fig. S2).

Analysis by hematologic response at 3 months per Mayo2004/European stage showed that patients with stage I or II disease achieving CR did not reach a median OS, while for patients with stages IIIa and IIIb, median OS was 56.2 and 47.0 months, respectively. The respective median OS for patients with stage I, II, IIIa, and IIIb disease achieving VGPR was 81.1, 111.6, 60.5 and 35.7 months, and 83.5, 70.2, 44.6, and 18.5 months for those with PR. Finally, patients failing to achieve a hematologic response by 3 months had a median OS of 96.4, 23.3, 8.9, and 3.3 months. The between-response difference in OS was statistically significant for all stages (*p* < 0.001 by log-rank; Fig. S3).

## Discussion

To the best of our knowledge, EMN23 is the largest study assessing real-word characteristics and outcomes of patients with systemic AL amyloidosis. The analysis of 4480 patient records provides a mosaic that is representative of the heterogeneity of this disease and the challenges associated with its management.

For AL amyloidosis, due to the difficulties associated with the diagnosis and management of the patients, a substantial proportion of them are treated in referral centers, often when their disease has advanced. At the same time, large-scale real-world data can only be collected from Sites with the relevant expertise, as these centers are usually the ones which have the required resources and specialized knowledge to perform systematic and reliable data collection.

Delayed diagnosis remains a major challenge in AL amyloidosis. Despite advances in diagnosis (introduction of serum free light chain measurement and the use of cardio-biomarkers), time to diagnosis did not improve, with half of the patients being diagnosed more than 5 months after the first symptoms, often at an advanced cardiac stage, with more than one third presenting with stage III disease. These observations strongly support the need to further increase disease awareness and invest in the screening of high-risk populations.

Early mortality remained high and essentially unchanged within more than 15 years of therapeutic advances and the introduction of bortezomib, reflecting the delayed diagnosis and the advanced cardiac disease at treatment initiation. These real-world outcomes differ from those published from tertiary referral centers in the United States, where improvements in survival have been observed [20, 21]. The more extensive use of ASCT in these experienced centers also indicates a more selected population.

An unmet need is to improve the outcomes of stage IIIb patients, in whom early mortality remains at 40%. New treatment strategies, beyond clonal-targeted therapy, are needed; however, early and deep hematologic response remains the most effective strategy and can improve survival (OS of stage IIIa and IIIb complete responders at 3 months was 56.2 and 47.0 months, respectively). Unfortunately, the optimal regimen for this frail population is yet to be developed. In contrast, the prognosis of stage I patients is good; these patients may also benefit from intensive therapies. ASCT-related mortality was low (3-month OS rate 97.8%) indicating that in low-risk and carefully selected patients, ASCT is also safe [28]. Patients with stage II and stage IIIa disease seem to benefit the most from new therapies; OS in this patient population improved after 2010 (although it did not reach statistical significance for stage II), probably due to the introduction of bortezomib. These patients are fitter and tolerate bortezomib better than stage IIIb patients, while they may still have reversible organ damage.

Bortezomib-based regimens were the most commonly used after 2010 and the variations (VCD, VD, and VMD) had similar efficacy. Responses to VCD and the other bortezomib-based regimens were lower than in clinical trials, probably due to the exclusion of frail, and of stage IIIb patients from prospective clinical trials [14, 16–18, 29]. These results highlight the importance of real-world data in the evaluation of treatments outside the strict context of clinical trials.

Importantly, first-line therapy, even with bortezomib-based regimens, was associated with primary failure in one in three patients and approximately half achieved deep hematologic responses. Management of patients, who do not respond to first-line therapy is challenging since available second-line options (such as IMiDs) show lower efficacy and are less well tolerated than in myeloma patients [13, 30]. Recent advances with the more extensive use of daratumumab or venetoclax in some selected patients have improved treatment options in the relapsed/refractory setting but, in our study, stage IIIb patients had lower probability to receive a salvage therapy: many died early or could not tolerate the available medication. Thus, for such patients it seems that there is only one chance to give an effective therapy and is therefore logical to use the most effective one at treatment initiation, without delay. The recently approved Daratumumab-VCD is associated with a significant improvement in CR and VGPR rates in patients with stage IIIa disease [18], but no data have been published in a journal so far for stage IIIb patients. The real-world efficacy of this new regimen remains also to be assessed.

Second-line therapy followed the evolution of myeloma therapies and the extensive use of bortezomib at first line. Class switch was a common strategy; re-use of successful therapies was also applied and of bortezomib in particular. To our knowledge such data have not been reported so far and are of clinical relevance, even though the treatment options continue to expand.

This real-world study highlighted the prognostic value of the currently used staging systems (modified Mayo 2004 and revised Mayo 2012), of current hematologic response criteria and of early evaluation of the hematologic responses [24]. To the best of our knowledge, this is the first real-world validation of the importance of rapid hematologic responses. Furthermore, the importance of rapid hematologic responses for patients with more advanced disease is emphasized, supporting the primary use of the most effective therapies and early regimen switch in non-responding patients.

Even though deeper hematologic responses are associated with improved survival, and despite the fact that these were more frequent and rapid in the post-2010 period, the overall survival did not seem to improve significantly. However, the benefit in survival post-2010 may be underestimated as potential improvements in the OS of stage I patients may be masked by the shorter follow-up time in the more recent cohort.

Limitations of the study include the heterogeneity of the cohort, lack of complete datasets for several patients, non-centrally evaluated hematologic response, change of response criteria over time (in 2005 and after 2012 [23, 24]), and difficulties in the assessment of organ responses. All these limitations are inherent to registry studies which depict the practice at the time of data recording, as well as the dynamic changes that occurred within 15 years. Nonetheless, real-world data provide a valuable guide for the design of clinical studies that evaluate therapies, highlight the deficiencies in specific aspects of disease management and can guide clinical research and the allocation of valuable health care resources.

This large real-world study captures the evolution of management of AL amyloidosis in Europe and provides valuable data on the outcomes of this rare disease. The results point to the unmet needs of early diagnosis, management of high-risk disease and of primary treatment failure. Based on the real-world evidence obtained from EMN23, efforts are required to improve early detection of AL amyloidosis; this can be realized through the intensification of education of the medical community to increase disease awareness, new biomarkers to identify high-risk groups, the design and evaluation of screening programs for high-risk populations, and the development of new diagnostic tools. Efforts are also required to develop safe but highly and rapidly effective anti-clonal therapies, while awaiting the results of studies that currently evaluate amyloid-targeting therapies.

## Supplementary information

Supplementary material

## Data Availability

All data mentioned in the main text is accompanied by the relevant tables or figures in the “Results” section or in the Supplementary material. Individual patient data will not be shared.
