# Supplementary material for: The management of light chain (AL) amyloidosis in Europe: clinical characteristics, treatment patterns, and efficacy outcomes between 2004 and 2018
Source: Blood Cancer J. 2023 Jan 25;13(1):19. doi: 10.1038/s41408-023-00789-8 (PMC9876983; doi:10.1038/s41408-023-00789-8)
Supplement: Supplementary file 1 — Supplementary material [file 41408_2023_789_MOESM1_ESM.docx]

# **Supplementary Material**

Article:
The Management of Light Chain (AL) Amyloidosis in Europe:
Clinical Characteristics, Treatment Patterns and Efficacy Outcomes between 2004 and 2018

**Contents**

**List of supplementary tables**

[**Table S1: Number of patients enrolled per country** 5](#_Toc122525393)

[**Table S2: IgM-related Amyloidosis, overall (2004–2018), and per treatment period** 6](#_Toc122525394)

[**Table S3: First-line treatment regimen groups by country, 2004–2018** 7](#_Toc122525395)

[**Table S4: First-line treatment regimens by Mayo2004/European cardiac stage and by first-line treatment initiation period** 8](#_Toc122525396)

[**Table S5: First-line treatment regimen group by organ involvement (2004-2018)** 9](#_Toc122525397)

[**Table S6: First-line treatment options for patients with IgM-related Amyloidosis, by first-line treatment regimen group, and by first line-treatment initiation period** 10](#_Toc122525398)

[**Table S7: Best hematologic responses, by line of treatment, 2004–2018** 11](#_Toc122525399)

[**Table S8: Best hematologic responses by first-line regimen group, 2004–2018** 12](#_Toc122525400)

[**Table S9: Best hematologic responses, by first-line individual regimen in 2004–2018** 13](#_Toc122525401)

[**Table S10: Best hematologic responses, by first-line regimen, and by first-line treatment initiation period** 14](#_Toc122525402)

[**Table S11: Best hematologic responses, by first-line treatment initiation period** 15](#_Toc122525403)

[**Table S12: Hematologic responses at 3 and 6 months, overall and by first-line treatment initiation period** 16](#_Toc122525404)

[**Table S13 Hematologic responses at 3 and 6 months, by first-line treatment regimen (2004–2018)** 17](#_Toc122525405)

[**Table S14: Median ToT, overall and by first-line treatment initiation period and cardiac stage** 18](#_Toc122525406)

[**Table S15: Second-line treatment options by first-line regimen group, and by first-line treatment initiation period** 19](#_Toc122525407)

[**Table S16: Median follow-up, overall and by first-line treatment initiation period** 20](#_Toc122525408)

[**Table S17: Time-to-Event outcomes, overall and by first-line treatment initiation period** 21](#_Toc122525409)

[**Table S18 Early mortality rates overall and by first-line treatment initiation period** 22](#_Toc122525410)

[**Table S19: Overall survival by first-line regimen group and by cardiac stage, 2004–2018** 23](#_Toc122525411)

[**Table S20: Time-to-event outcomes for IgM-patients, overall and by first-line treatment initiation period** 24](#_Toc122525412)

**List of supplementary figures**

[**Figure S1: Kaplan-Meier graph of overall survival (OS) by Mayo2012 revised cardiac stage at diagnosis** 24](#_Toc122525413)

[**Figure S2: Kaplan-Meier graph of OS by hematologic response at 6 months after first-line treatment initiation, 2004–2018** 25](#_Toc122525414)

[**Figure S3: Kaplan-Meier graph of OS by hematologic response at 3 months after first-line treatment initiation, and by cardiac stage, 2004−2018** 26](#_Toc122525415)

**Methodology**

Hematologic responses were assessed by the Investigators and according to the consensus criteria. However, as per known limitations associated with the design of retrospective studies, risk of reduced uniform staging and response criteria between sites is more likely to have happened compared to a clinical trial setting.

OS for each patient was calculated as the time (months) from treatment start until the date of death, after applying the following imputations: patients who were still alive on 31 December 2018 were censored on 31 December 2018; patients who were alive at the last contact were censored at the maximum date (before 31 December 2018) that it was known that they were alive. In cases where a patient started treatment and died during the same month, OS was set to one month.

Incomplete data (i.e., missing or partially unknown treatment start and stop dates, unknown treatment outcome without initiating any subsequent line of treatment) were excluded from the analysis of ToT. Patients who received induction therapy before ASCT, within the study period, but had ASCT after 31 December 2018, were categorized as “ASCT”; however, these patients were excluded from the time-to-event analyses. For patients who started a second line therapy, the time to second-line therapy was calculated from the date of initiation of first-line therapy until the date of initiation for of second line therapy.

**Tables**

**Table S1: Number of patients enrolled per country**

| **Country** | **Site** | **Number of patients** |
| --- | --- | --- |
| Austria | Department of Internal Medicine I, Division of Oncology, Medical University Vienna | 68 |
| Czech Republic | Department of Hematooncology, University Hospital Ostrava | 18 |
| France | - National Amyloidosis Center and Hematology Unit, CHU Limoges - Nephrology Unit, CHU Poitiers | 290 |
| Germany | Medical Department V, Amyloidosis Center, Heidelberg University Hospital | 502 |
| Greece | Department of Clinical Therapeutics, School of Medicine, National and Kapodistrian University of Athens | 256 |
| Italy | Amyloidosis Research and Treatment Center, Fondazione IRCCS Policlinico San Matteo, Department of Molecular Medicine, University of Pavia | 1,348 |
| The Netherlands | - Amyloidosis Center of Expertise Department of Internal Medicine, Faculty of Medical Sciences, University Medical Center Groningen - Department of Hematology, University Medical Center Utrecht | 165 |
| Portugal | - Department of Hematology, Hospital São João, Porto - Hematology Department, Champalimaud Center for the Unknown, Lisbon | 20 |
| Spain | Amyloidosis and Myeloma Unit, Department of Hematology, Hospital Clínic, Barcelona | 123 |
| UK | National Amyloidosis Centre, University College London, London | 1,690 |

**Table S2: IgM-related Amyloidosis, overall (2004–2018), and per treatment period**

|  |  | **2004–2018, N (%)** | **Pre-2010, N (%)** | **Post-2010, N (%)** |
| --- | --- | --- | --- | --- |
| Patients who initiated first-line treatment |  | 4,480 | 1,415 | 3,065 |
| Serum: IgM-Amyloidosis | Yes | 197 (4.4) | 69 (4.9) | 128 (4.2) |
|  | No | 4283 (95.6) | 1346 (95.1) | 2937 (95.8) |
| Urine: IgM-Amyloidosis | Yes | 4 (0.1) | 2 (0.1) | 2 (0.1) |
|  | No | 4476 (99.9) | 1413 (99.9) | 3063 (99.9) |

**Table S3: First-line treatment regimen groups by country, 2004–2018**

| **Response** |  | **Greece, N (%)** | **Portugal, N (%)** | **Czech Republic, N (%)** | **Austria, N (%)** | **Netherlands, N (%)** | **France, N (%)** | **Germany, N (%)** | **Italy, N (%)** | **Spain, N (%)** | **UK, N (%)** | |
| --- | --- | --- | --- | --- | --- | --- | --- | --- | --- | --- | --- | --- |
|  | | | | | | | | | | | |  |
| **Patients** | N | 256 | 20 | 18 | 68 | 165 | 290 | 502 | 1,348 | 123 | 1,690 | |
| **Regimen groups** | Bor-based | 118 (46.1%) | 12 (60.0%) | 4 (22.2%) | 31 (45.6%) | 51 (30.9%) | 120 (41.4%) | 306 (61%) | 660 (49%) | 29 (23.6%) | 1123 (66.4%) | |
|  | IMiD-based | 12 (4.7%) | 2 (10.0%) |  | 1 (1.5%) | 20 (12.1%) | 3 (1%) | 14 (2.8%) | 131 (9.7%) | 1 (0.8%) | 304 (18%) | |
|  | Chemo | 21 (8.2%) | 2 (10.0%) | 3 (16.7%) | 1 (1.5%) | 15 (9.1%) | 124 (42.8%) | 88 (17.5%) | 426 (31.6%) | 30 (24.4%) | 159 (9.4%) | |
|  | Rituximab-based | 2 (0.8%) |  |  | 4 (5.9%) | 9 (5.5%) | 21 (7.2%) | 7 (1.4%) | 41 (3.0%) |  | 11 (0.7%) | |
|  | Dara-based | 1 (0.4%) |  |  | 14 (20.6%) |  | 1 (0.3%) | 5 (1.0%) |  |  |  | |
|  | ASCT | 18 (7.0%) | 4 (20.0%) | 7 (38.9%) | 10 (14.7%) | 41 (24.8%) | 2 (0.7%) | 39 (7.8%) | 23 (1.7%) | 43 (35%) | 91 (5.4%) | |
|  | Steroids | 1 (0.4%) |  | 1 (5.6%) |  | 1 (0.6%) |  | 1 (0.2%) | 43 (3.2%) |  | 2 (0.1%) | |
|  | Clinical Trial | 82 (32.0%) |  | 3 (16.7%) | 6 (8.8%) | 17 (10.3%) | 16 (5.5%) | 9 (1.8%) | 24 (1.8%) | 20 (16.3%) |  | |
|  | Other | 1 (0.4%) |  |  | 1 (1.5%) | 11 (6.7%) | 3 (1.0%) | 33 (6.6%) |  |  |  | |

The total number of patients in this analysis is 4480. Percentages are calculated using the total number of patients in each column (country) as the denominator.

ASCT=autologous stem cell transplantation; Bor=bortezomib; Chemo=chemotherapy; Dara=daratumumab; IMiD= immunomodulatory drugs; N=number of patients

**Table S4: First-line treatment regimens by Mayo2004/European cardiac stage** **and by first-line treatment initiation period**

|  | **Stage I** | | **Stage II** | | **Stage IIIa** | | **Stage IIIb** | | **Unknown** | |
| --- | --- | --- | --- | --- | --- | --- | --- | --- | --- | --- |
| **Period^*^** | **Pre-2010** | **Post-2010** | **Pre-2010** | **Post-2010** | **Pre-2010** | **Post-2010** | **Pre-2010** | **Post-2010** | **Pre-2010** | **Post-2010** |
| **Patients, N** | 184 | 512 | 347 | 1066 | 150 | 853 | 178 | 485 | 556 | 149 |
| **Regimen group, N (%)**^§^ | | | | | | | | | | |
| Bor-based | 23 (12.5) | 332 (64.8) | 53 (15.3) | 780 (73.2) | 32 (21.3) | 689 (80.8) | 36 (20.2) | 399 (82.3) | 19 (3.4) | 91 (61.1) |
| IMiD-based | 27 (14.7) | 11 (2.1) | 72 (20.7) | 16 (1.5) | 19 (12.7) | 23 (2.7) | 54 (30.3) | 7 (1.4) | 257 (46.2) | 2 (1.3) |
| Chemo | 73 (39.7) | 43 (8.4) | 171 (49.3) | 100 (9.4) | 76 (50.7) | 66 (7.7) | 76 (42.7) | 43 (8.9) | 207 (37.2) | 14 (9.4) |
| Rituximab-based | 7 (3.8) | 20 (3.9) | 9 (2.6) | 21 (2.0) | 2 (1.3) | 8 (0.9) | 3 (1.7) | 6 (1.2) | 8 (1.4) | 11 (7.4) |
| Dara-based | - | 1 (0.2) | - | 5 (0.5) | - | 10 (1.2) | - | 5 (1.0) | - | - |
| Steroids | 9 (4.9) | 2 (0.4) | 13 (3.7) | 4 (0.4) | 5 (3.3) | 2 (0.2) | 3 (1.7) | - | 8 (1.4) | 3 (2.0) |
| ASCT | 33 (17.9) | 74 (14.5) | 17 (4.9) | 47 (4.4) | 5 (3.3) | 24 (2.8) | 1 (0.6) | 6 (1.2) | 52 (9.4) | 19 (12.8) |
| Clinical trial | 11 (6.0) | 23 (4.5) | 9 (2.6) | 82 (7.7) | 8 (5.3) | 24 (2.8) | 3 (1.7) | 9 (1.9) | 4 (0.7) | 4 (2.7) |
| Other regimen groups | 1 (0.5) | 6 (1.2) | 3 (0.9) | 11 (1.0) | 3 (2.0) | 7 (0.8) | 2 (1.1) | 10 (2.1) | 1 (0.2) | 5 (3.4) |
| **Individual regimens, N (%)**^§¶^ | | | | | | | | | | |
| VCD | 4 (2.2) | 98 (19.1) | 5 (1.4) | 218 (20.5) | 4 (2.7) | 167 (19.6) | 6 (3.4) | 93 (19.2) | 4 (0.7) | 54 (36.2) |
| MDex | 66 (35.9) | 39 (7.6) | 148 (42.7) | 98 (9.2) | 69 (46) | 57 (6.7) | 69 (38.8) | 43 (8.9) | 106 (19.1) | 12 (8.1) |
| VMD | 5 (2.7) | 21 (4.1) | 20 (5.8) | 66 (6.2) | 7 (4.7) | 39 (4.6) | 9 (5.1) | 34 (7) | 3 (0.5) | 6 (4) |
| RD | 1 (0.5) | 4 (0.8) | 4 (1.2) | 3 (0.3) | - | 5 (0.6) | 2 (1.1) | 4 (0.8) | 1 (0.2) | 1 (0.7) |
| VD | 11 (6.0) | 35 (6.8) | 24 (6.9) | 93 (8.7) | 18 (12) | 82 (9.6) | 16 (9) | 88 (18.1) | 10 (1.8) | 24 (16.1) |
| CTD | 20 (10.9) | 2 (0.4) | 44 (12.7) | 8 (0.8) | 7 (4.7) | 8 (0.9) | 32 (18) | 2 (0.4) | 234 (42.1) | - |
| ASCT | 33 (17.9) | 74 (14.5) | 17 (4.9) | 47 (4.4) | 5 (3.3) | 24 (2.8) | 1 (0.6) | 6 (1.2) | 52 (9.4) | 19 (12.8) |
| Clinical trial | 11 (6.0) | 23 (4.5) | 9 (2.6) | 82 (7.7) | 8 (5.3) | 24 (2.8) | 3 (1.7) | 9 (1.9) | 4 (0.7) | 4 (2.7) |
| Other individual regimens | 33 (17.9) | 216 (42.2) | 76 (21.9) | 451 (42.3) | 32 (21.3) | 447 (52.4) | 40 (22.5) | 206 (42.5) | 142 (25.5) | 29 (19.5) |

^*^Pre- and post-2010 indicate the periods of 2004-2010 and 2011-2018, respectively. ^§^Percentages are calculated using the total number of patients in each column as the denominator. ^¶^Individual regimen data were not reported for UK patients. ASCT=autologous stem cell transplantation; Bor=bortezomib; C=cyclophosphamide; Chemo=chemotherapy; D, Dex=dexamethasone; Dara=daratumumab; IMiD= immunomodulatory drugs; M=melphalan; N=number of patients; R=lenalidomide; T=thalidomide; V=bortezomib

**Table S5: First-line treatment regimen group by organ involvement (2004-2018)**

|  |  | **Total** | **IMiD-based** | **Bor-based** | **Chemo** | **Rituximab-based** | **Dara-based** | **ASCT** | **Steroids** | **Clinical Trial** | **Other** | |  |
| --- | --- | --- | --- | --- | --- | --- | --- | --- | --- | --- | --- | --- | --- |
|  | | | | | | | | | | | |  | |
| **Organ involvement** | Kidney | 2,961 | 309 (10.4) | 1627 (54.9) | 569 (19.2) | 57 (1.9) | 11 (0.4) | 195 (6.6) | 40 (1.4) | 125 (4.2) | 28 (0.9) | |  |
|  | Heart | 3,041 | 326 (10.7) | 1747 (57.4) | 600 (19.7) | 46 (2.5) | 18 (0.6) | 115 (3.8) | 23 (0.8) | 127 (4.2) | 39 (1.3) | |  |
|  | Liver | 640 | 70 (10.9) | 334 (52.2) | 131 (20.5) | 17 (2.7) | 2 (0.3) | 39 (6.1) | 13 (2.0) | 27 (4.2) | 7 (1.1) | |  |
|  | Nervous system | 646 | 48 (7.4) | 284 (44.0) | 206 (31.9) | 9 (1.4) | 6 (0.9) | 40 (6.2) | 8 (1.2) | 34 (5.3) | 11 (1.7) | |  |
|  | Gastrointestinal tract | 283 | 12 (4.2) | 135 (47.7) | 73 (25.8) | 9 (3.2) | 4 (1.4) | 21 (7.4) | 3 (1.1) | 20 (7.1) | 6 (2.1) | |  |
|  | Lung | 39 | 4 (10.3) | 10 (25.6) | 9 (23.1) | 6 (15.4) | 0 (0.0) | 6 (15.4) | 1 (2.6) | 2 (5.1) | 1 (2.6) | |  |
|  | Soft tissue | 802 | 43 (5.4) | 465 (58.0) | 152 (19.0) | 20 (2.5) | 4 (0.5) | 58 (7.2) | 5 (0.6) | 36 (4.5) | 19 (2.4) | |  |
| ASCT=autologous stem cell transplantation; Bor=bortezomib; Chemo=chemotherapy; Dara=daratumumab; IMiD= immunomodulatory drugs | | | | | | | | | | | |  | |

**Table S6: First-line treatment options for patients with IgM-related Amyloidosis, by first-line treatment regimen group, and by first line-treatment initiation period**

|  |  | **2004–2018,**  **N (%)** | **Pre-2010,**  **N (%)** | **Post-2010,**  **N (%)** |
| --- | --- | --- | --- | --- |
| **Patients who initiated first-line treatment** |  | 197 | 69 | 128 |
| **Regimen group** | IMiD-based | 5 (2.5) | 4 (5.8) | 1 (0.8) |
|  | Bor-based | 61 (31.0) | 4 (5.8) | 57 (44.5) |
|  | Chemo | 46 (23.4) | 34 (49.3) | 12 (9.4) |
|  | Rituximab-based | 74 (37.6) | 23 (33.3) | 51 (39.8) |
|  | Dara-based | 1 (0.5) |  | 1 (0.8) |
|  | ASCT | 5 (2.5) | 4 (5.8) | 1 (0.8) |
|  | Clinical Trial | 4 (2.0) |  | 4 (3.1) |
|  | Other | 1 (0.5) |  | 1 (0.8) |
| **Individual regimen**^¶^ | ASCT | 5 (2.5) | 4 (5.8) | 1 (0.8) |
|  | VCD | 12 (6.1) | 1 (1.4) | 11 (8.6) |
|  | MDex | 27 (13.7) | 18 (26.1) | 9 (7.0) |
|  | VMD | 2 (1.0) | 1 (1.4) | 1 (0.8) |
|  | VD | 4 (2.0) | 1 (1.4) | 3 (2.3) |
|  | CTD | 4 (2.0) | 3 (4.3) | 1 (0.8) |
|  | Clinical trial | 4 (2.0) |  | 4 (3.1) |
|  | Other | 139 (70.6) | 41 (59.4) | 98 (76.6) |
| Note: (%) = (number of patients/period’s population) x100. Pre- and post-2010 indicate the periods of 2004-2010 and 2011-2018, respectively. ^¶^Percentages do not add-up to 100.0% as not all treatment options are presented. ASCT=autologous stem cell transplantation; Bor=bortezomib; C=cyclophosphamide; Chemo=chemotherapy; D, Dex=dexamethasone; Dara=daratumumab; IMiD= immunomodulatory drugs; M=melphalan; N=number of patients; R=lenalidomide; T=thalidomide; V=bortezomib | | | | |

**Table S7: Best hematologic responses, by line of treatment, 2004–2018**

|  | **2004-2018, N (%) 1^st^ line** | **2004-2018, N (%) 2^nd^ line** |
| --- | --- | --- |
| **Patients** | 4,480 | 1,759 |
| **Patients with response assessment** | 3,952 (88.2) | 1,013 (57.6) |
| NA | 351 (7.8) | 708 (40.2) |
| Clinical trial | 177 (4.0) | 38 (2.2) |
| **Best hematologic response** |  |  |
| CR | 916 (23.2) | 155 (15.3%) |
| VGPR | 836 (21.2) | 204 (20.1%) |
| PR | 752 (19.0) | 236 (23.3%) |
| SD | 609 (15.4) | 243 (24.0%) |
| PD | 502 (12.7) | 82 (8.1%) |
| Death prior to 3 months | 337 (8.5) | 93 (9.2%) |

Best hematologic response percentages are calculated using the number of patients with available response assessment and patients without response data who died prior to 3 months. The category “death prior to 3 months” refers to patients without available response data, who died within 3 months from first-line treatment initiation. CR=complete response; NA=not available; PD=progressive disease; PR=partial response; SD=stable disease; VGPR=very good partial response.

**Table S8: Best hematologic responses by first-line regimen group, 2004–2018**

|  | **IMiD-based** | **Bor-based** | **Chemo** | **Rituximab-based** | **Dara-based** | **ASCT** | **Steroids** | **Other** |  |
| --- | --- | --- | --- | --- | --- | --- | --- | --- | --- |
| **Patients, N** | 488 | 2,454 | 869 | 95 | 21 | 278 | 49 | 49 |  |
| **Patients with response assessment, N (%)** | 464 (95.1) | 2,275 (92.7) | 762 (87.7) | 79 (83.2) | 20 (95.2) | 267 (96.0) | 43 (87.8) | 42 (85.7) |  |
| **NA, N (%)** | 24 (4.9) | 179 (7.3) | 107 (12.3) | 16 (16.8) | 1 (4.8) | 11 (4.0) | 6 (12.2) | 7 (14.3) |  |
| **Best hematologic response, N (%)** | | | | | | | | | |
| CR | 81 (17.5) | 593 (26.1) | 109 (14.3) | 2 (2.5) | 4 (20.0) | 116 (43.4) | 8 (18.6) | 3 (7.1) |  |
| VGPR | 43 (9.3) | 585 (25.7) | 102 (13.4) | 13 (16.5) | 8 (40.0) | 72 (27.0) | 7 (16.3) | 6 (14.3) |  |
| PR | 125 (26.9) | 378 (16.6) | 162 (21.3) | 25 (31.6) | 3 (15.0) | 47 (17.6) | 4 (9.3) | 8 (19.0) |  |
| SD | 108 (23.3) | 340 (14.9) | 116 (15.2) | 16 (20.3) | 3 (15.0) | 19 (7.1) | 1 (2.3) | 6 (14.3) |  |
| PD | 67 (14.4) | 193 (8.5) | 188 (24.7) | 17 (21.5) |  | 10 (3.7) | 20 (46.5) | 7 (16.7) |  |
| Death prior to 3 months | 40 (8.6) | 186 (8.2) | 85 (11.2) | 6 (7.6) | 2 (10.0) | 3 (1.1) | 3 (7.0) | 12 (28.6) |  |

Best hematologic response percentages are calculated using the number of patients with available response assessment and patients without response data who died prior to 3 months. The category “death prior to 3 months” refers to patients without available response data, who died within 3 months from first-line treatment initiation.  ASCT=autologous stem cell transplantation; Bor=bortezomib; Chemo=chemotherapy; CR=complete response; IMiD=immunomodulatory drug; N=number of patients; NA=not available; PD=progressive disease; PR=partial response; SD=stable disease; VGPR=very good partial response.

**Table S9: Best hematologic responses, by first-line individual regimen in 2004–2018**

|  | **VCD** | **MDex** | **VD** | **VMD** | **ASCT** | **CTD** | **RD** |  |
| --- | --- | --- | --- | --- | --- | --- | --- | --- |
| **Patients, N** | 653 | 707 | 401 | 210 | 278 | 357 | 25 |  |
| **Patients with response assessment, N (%)** | 567 (86.8) | 606 (85.7) | 354 (88.3) | 171 (81.4) | 267 (96.0) | 348 (97.5) | 24 (96.0) |  |
| **NA, N (%)** | 86 (13.2) | 101 (14.3) | 47 (11.7) | 39 (18.6) | 11 (4.0) | 9 (2.5) | 1 (4.0) |  |
| **Best hematologic response, N (%)** | | | | | | | | |
| CR | 109 (19.2) | 92 (15.2) | 45 (12.7) | 35 (20.5) | 116 (43.4) | 72 (20.7) | 1 (4.2) |  |
| VGPR | 157 (27.7) | 92 (15.2) | 97 (27.4) | 43 (25.1) | 72 (27.0) | 28 (8.0) | 3 (12.5) |  |
| PR | 95 (16.8) | 125 (20.6) | 78 (22.0) | 20 (11.7) | 47 (17.6) | 104 (29.9) | 6 (25.0) |  |
| SD | 16 (2.8) | 60 (9.9) | 55 (15.5) | 1 (0.6) | 19 (7.1) | 86 (24.7) | 5 (20.8) |  |
| PD | 102 (18.0) | 158 (26.1) | 30 (8.5) | 28 (16.4) | 10 (3.7) | 48 (13.8) | 4 (16.7) |  |
| Death prior to 3 months | 88 (15.5) | 79 (13.0) | 49 (13.8) | 44 (25.7) | 3 (1.1) | 10 (2.9) | 5 (20.8) |  |

Best hematologic response percentages are calculated using the number of patients with available response assessment and patients without response data who died prior to 3 months. The category “death prior to 3 months” refers to patients without available response data, who died within 3 months from first-line treatment initiation. Not all individual regimens are presented.

ASCT=autologous stem cell transplantation; C=cyclophosphamide; CR=complete response; D, Dex=dexamethasone; M=melphalan; N=number of patients; NA=not available; PD=progressive disease; PR=partial response; R=lenalidomide; SD=stable disease; T=thalidomide; V=bortezomib; VGPR=very good partial response.

**Table S10: Best hematologic responses, by first-line regimen, and by first-line treatment initiation period**

| **Pre-2010** | **IMiD-based** | **Bor-based** | **Chemo** | **Rituximab-based** | **Dara-based** | **ASCT** | **Steroids** | **Other** |
| --- | --- | --- | --- | --- | --- | --- | --- | --- |
|  |  |  |  |  |  |  |  |  |
| **Patients, N** | 429 | 163 | 603 | 29 | 0 | 108 | 38 | 10 |
| **Patients with response assessment, N (%)** | 412 (96.0) | 146 (89.6) | 553 (91.7) | 29 (100.0) | 0 | 105 (97.2) | 35 (92.1) | 9 (90.0) |
| **NA, N (%)** | 17 (4.0) | 17 (10.4) | 50 (8.3) | 0 (0.0) | 0 | 3 (2.8) | 3 (7.9) | 1 (10.0) |
| **Best hematologic response, N (%)^*^** |  |  |  |  |  |  |  |  |
| CR | 78 (18.9) | 41 (28.1) | 88 (15.9) | 1 (3.4) | - | 34 (32.4) | 7 (20.0) | 2 (22.2) |
| VGPR | 37 (9.0) | 26 (17.8) | 63 (11.4) | 5 (17.2) | - | 22 (21.0) | 5 (14.3) | 2 (22.2) |
| PR | 116 (28.2) | 27 (18.5) | 123 (22.2) | 12 (41.4) | - | 23 (21.9) | 2 (5.7) | 3 (33.3) |
| SD | 100 (24.3) | 15 (10.3) | 78 (14.1) | 6 (20.7) | - | 15 (14.3) | 1 (2.9) | 0 (0.0) |
| PD | 52 (12.6) | 15 (10.3) | 153 (27.7) | 3 (10.3) | - | 9 (8.6) | 18 (51.4) | 2 (22.2) |
| Death prior to 3 months | 29 (7.0) | 22 (15.1) | 48 (8.7) | 2 (6.9) | - | 2 (1.9) | 2 (5.7) | 0 (0.0) |
| **Post-2010** | **IMiD-based** | **Bor-based** | **Chemo** | **Rituximab-based** | **Dara-based** | **ASCT** | **Steroids** | **Other** |
| **Patients, N** | 59 | 2,291 | 266 | 66 | 21 | 170 | 11 | 39 |
| **Patients with response assessment, N (%)** | 52 (88.1) | 2,129 (92.9) | 209 (78.6) | 50 (75.8) | 20 (95.2) | 162 (95.3) | 8 (72.7) | 33 (84.6) |
| **NA, N (%)** | 7 (11.9) | 162 (7.1) | 57 (21.4) | 16 (24.2) | 1 (4.8) | 8 (4.7) | 3 (27.3) | 6 (15.4) |
| **Best hematologic response, N (%)^*^** |  |  |  |  |  |  |  |  |
| CR | 3 (5.8) | 552 (25.9) | 21 (10.0) | 1 (2.0) | 4 (20.0) | 82 (50.6) | 1 (12.5) | 1 (3.0) |
| VGPR | 6 (11.5) | 559 (26.3) | 39 (18.7) | 8 (16.0) | 8 (40.0) | 50 (30.9) | 2 (25.0) | 4 (12.1) |
| PR | 9 (17.3) | 351 (16.5) | 39 (18.7) | 13 (26.0) | 3 (15.0) | 24 (14.8) | 2 (25.0) | 5 (15.2) |
| SD | 8 (15.4) | 325 (15.3) | 38 (18.3) | 10 (20.0) | 3 (15.0) | 4 (2.5) | - | 6 (18.2) |
| PD | 15 (28.8) | 178 (8.4) | 35 (16.7) | 14 (28.0) | - | 1 (0.6) | 2 (25.0) | 5 (15.2) |
| Death prior to 3 months | 11 (21.2) | 164 (7.7) | 37 (17.7) | 4 (8.0) | 2 (10.0) | 1 (0.6) | 1 (12.5) | 12 (36.4) |
|  |  |  |  |  |  |  |  |  |

^*^Best hematologic response percentages are calculated using the number of patients with available response assessment and patients without response data who died prior to 3 months. The category “death prior to 3 months” refers to patients without available response data, who died within 3 months from first-line treatment initiation. ASCT=autologous stem cell transplantation; CR=complete response; N=number of patients; NA=not available; PD=progressive disease; PR=partial response; SD=stable disease; VGPR=very good partial response.

**Table S11: Best hematologic responses, by first-line treatment initiation period**

|  | **Pre-2010, N (%)** | **Post-2010, N (%)** |
| --- | --- | --- |
| **Patients who initiated 1st line** | 1,415 | 3,065 |
| **Patients with response assessment** | 1,289 (91.1) | 2,663 (86.9) |
| **NA or participating in clinical trial** | 126 (8.9) | 402 (13.1) |
| **Best hematologic response*** |  |  |
| CR | 251 (19.5) | 665 (25.0) |
| VGPR | 160 (12.4) | 676 (25.4) |
| PR | 306 (23.7) | 446 (16.7) |
| SD | 215 (16.7) | 394 (14.8) |
| PD | 252 (19.6) | 250 (9.4) |
| Death prior to 3 months | 105 (8.1) | 232 (8.7) |
| NA | 91 (6.4) | 260 (8.5) |
| Clinical trial | 35 (2.5) | 142 (4.6) |

*Best hematologic response percentages are calculated using the number of patients with available response assessment and patients without response data who died prior to 3 months. The category “death prior to 3 months” refers to patients without available response data, who died within 3 months from first-line treatment initiation. CR=complete response; N=number of patients; NA=not available; PD=progressive disease; PR=partial response; SD=stable disease; VGPR=very good partial response.

**Table S12: Hematologic responses at 3 and 6 months, overall and by first-line treatment initiation period**

|  | **2004–2018, N (%)** | **Pre-2010, N (%)** | **Post-2010, N (%)** |
| --- | --- | --- | --- |
| **Patients who initiated 1st line, N** | 4,480 | 1,415 | 3,065 |
| **Patients with response assessment at 3 months, N (%)** | 2,720 (60.7) | 754 (53.3) | 1,966 (64.1) |
| Not available | 1760 (39.3) | 661 (46.7) | 1099 (35.9) |
| **Hematologic response at 3 months from treatment initiation^*^** |  |  |  |
| CR | 447 (16.4) | 105 (13.9) | 342 (17.4) |
| VGPR | 505 (18.6) | 82 (10.9) | 423 (21.5) |
| PR | 642 (23.6) | 157 (20.8) | 485 (24.7) |
| No response (PD, SD) | 1126 (41.4) | 410 (54.4) | 716 (36.4) |
| **Patients with response assessment at 6 months, N (%)** | 2,329 (52.0) | 696 (49.2) | 1,633 (53.3) |
| Not available | 2151 (48.0) | 719 (50.8) | 1432 (46.7) |
| **Hematologic response at 6 months from treatment initiation^*^** |  |  |  |
| CR | 569 (24.4) | 134 (19.3) | 435 (26.6) |
| VGPR | 537 (23.1) | 82 (11.8) | 455 (27.9) |
| PR | 527 (22.6) | 190 (27.3) | 337 (20.6) |
| No response (PD, SD) | 696 (29.9) | 290 (41.7) | 406 (24.9) |

^*^Percentages are calculated using the number of patients with response assessment in each column as the denominator. . ASCT=autologous stem cell transplantation; CR=complete response; N=number of patients; NA=not available; PD=progressive disease; PR=partial response; SD=stable disease; VGPR=very good partial response.

**Table S13 Hematologic responses at 3 and 6 months, by first-line treatment regimen (2004–2018)**

|  | **IMiD-based** | **Bor-based** | **Chemo** | **Rituximab-based** | **Dara-based** | **ASCT** | **Steroids** | **Other** |  |
| --- | --- | --- | --- | --- | --- | --- | --- | --- | --- |
| **Patients, N** | 488 | 2,454 | 869 | 95 | 21 | 278 | 49 | 49 |  |
| **Patients with response assessment at 3 months, N (%)** | 325 (66.6) | 1679 (68.4) | 374 (43.0) | 52 (54.7) | 18 (85.7) | 235 (84.5) | 11 (22.4) | 26 (53.1) |  |
| Not available | 163 (33.4) | 775 (31.6) | 495 (57.0) | 43 (45.3) | 3 (14.3) | 43 (15.5) | 38 (77.6) | 23 (46.9) |  |
| **Response at 3 months from treatment initiation**^*^ | | | | | | | | | |
| CR | 46 (14.2) | 323 (19.2) | 33 (8.8) | 0 | 3 (16.7) | 41 (17.4) | 1 (9.1) | 0 |  |
| VGPR | 33 (10.2) | 352 (21.0) | 34 (9.1) | 4 (7.7) | 7 (38.9) | 71 (30.2) | 0 | 4 (15.4) |  |
| PR | 60 (18.5) | 401 (23.9) | 91 (24.4) | 14 (26.9) | 3 (16.7) | 64 (27.2) | 1 (9.1) | 8 (30.8) |  |
| No response (PD, SD) | 186 (57.2) | 603 (35.9) | 216 (57.8) | 34 (65.4) | 5 (27.8) | 59 (25.1) | 9 (81.8) | 14 (53.8) |  |
| **Patients with response assessment at 3 months, N (%)** | 316 (64.8) | 1422 (57.9) | 303 (34.9) | 39 (41.1) | 10 (47.6) | 212 (76.3) | 9 (18.4) | 18 (36.7) |  |
| Not available | 172 (35.2) | 1032 (42.1) | 566 (65.1) | 56 (58.9) | 11 (52.4) | 66 (23.7) | 40 (81.6) | 31 (63.3) |  |
| **Response at 6 months from treatment initiation**^*^ | | | | | | | | | |
| CR | 60 (19.0) | 394 (27.7) | 41 (13.5) | 0 | 3 (30.0) | 69 (32.5) | 1 (11.1) | 1 (5.6) |  |
| VGPR | 26 (8.2) | 387 (27.2) | 36 (11.9) | 7 (17.9) | 5 (50.0) | 71 (33.5) | 1 (11.1) | 4 (22.2) |  |
| PR | 88 (27.8) | 278 (19.5) | 87 (28.7) | 13 (33.3) | 1 (10.0) | 51 (24.1) | 1 (11.1) | 8 (44.4) |  |
| No response (PD, SD) | 142 (44.9) | 363 (25.5) | 139 (45.9) | 19 (48.7) | 1 (10.0) | 21 (9.9) | 6 (66.7) | 5 (27.8) |  |

^*^Percentages are calculated using the number of patients with response assessment in each column as the denominator. CR=complete response; N=number of patients; PD=progressive disease; PR=partial response; SD=stable disease; VGPR=very good partial response.

**Table S14: Median ToT, overall and by first-line treatment initiation period and cardiac stage**

|  | **2004–2018, N (%)** | **Pre-2010, N (%)** | **Post-2010, N (%)** |
| --- | --- | --- | --- |
| **All patients:** Total number of patients (Events - Censored) | 3742 (3621–121) | 1232 (1232–0) | 2510 (2389–121) |
| **Median ToT,** months (95% CI) | 4.72 (4.36–4.95) | 4.72 (4.23–4.95) | 4.79 (4.30–4.95) |
|  |  |  |  |
| **Stage I: N**umber of patients (Events – Censored) | 586 (566–20) | 154 (154–0) | 432 (412–20) |
| **Median ToT** in months (95% CI) | **5.67 (5.02–5.93)** | **5.51 (4.98–6.92)** | **5.80 (5.02–5.93)** |
|  |  |  |  |
| **Stage II**: number of patients (Events – Censored) | 1097 (1054–43) | 278 (278–0) | 819 (776–43) |
| **Median ToT** in months (95% CI) | **5.02 (4.95–5.08)** | **4.95 (4.23–5.70)** | **5.02 (4.98–5.05)** |
|  |  |  |  |
| **Stage IIIa:** number of patients (Events – Censored) | 846 (805–41) | 117 (117–0) | 729 (688–41) |
| **Median ToT** in months (95% CI) | **4.66 (4.03–4.95)** | **3.97 (2.89–4.82)** | **4.95 (4.03–4.98)** |
|  |  |  |  |
| **Stage IIIb:** number of patients (Events – Censored) | 574 (560–14) | 156 (156–0) | 418 (404–14) |
| **Median ToT** in months (95% CI) | **2.59 (2.36–2.95)** | **2.49 (2.16–3.11)** | **2.69 (2.36–2.95)** |

CI=Confidence interval; ToT=Time on treatment

**Table S15: Second-line treatment options by first-line regimen group, and by first-line treatment initiation period**

|  |  | **Second-line regimen groups^¶^** | | | |
| --- | --- | --- | --- | --- | --- |
| **First-line regimen groups, pre-2010**^§^ | **Patients, N** | **IMiD-based** | **Bor-based** | **Chemo** | **ASCT** |
| IMiD-based | 215 | 29 (13.5) | 133 (61.9) | 37 (17.2) | 10 (4.7) |
| Bor-based | 65 | 28 (43.1) | 16 (24.6) | 15 (23.1) | 3 (4.6) |
| Chemo | 364 | 143 (39.3) | 140 (38.5) | 59 (16.2) | 9 (2.5) |
| Rituximab-based | 11 | 2 (18.2) | 3 (27.3) | - | - |
| ASCT | 69 | 19 (27.5) | 33 (47.8) | 4 (5.8) | 7 (10.1) |
| Steroids | 30 | 8 (26.7) | 11 (36.7) | 5 (16.7) | 3 (10.0) |
| Clinical trial | 18 | - | 13 (72.2) | 3 (16.7) | - |
| Other regimen groups | 3 | - | - | 1 (33.3) |  |
|  |  | **Second-line regimen groups^§¶^** | | | |
| **First-line regimen groups, post-2010**^§^ | **Patients, N** | **IMiD-based** | **Bor-based** | **Chemo** | **ASCT** |
| IMiD-based | 25 | 6 (24.0) | 9 (36.0) | 4 (16.0) | 1 (4.0) |
| Bor-based | 692 | 333 (48.1) | 102 (14.7) | 86 (12.4) | 80 (11.6) |
| Chemo | 99 | 26 (26.3) | 54 (54.5) | 8 (8.1) | - |
| Rituximab-based | 33 | 4 (12.1) | 3 (9.1) | 7 (21.2) | - |
| Dara-based | 8 | - | - | 1 (12.5) | - |
| ASCT | 53 | 22 (41.5) | 4 (7.5) | 4 (7.5) | 14 (26.4) |
| Steroids | 2 | - | 2 (100.0) | - | - |
| Clinical Trial | 64 | 15 (23.4) | 25 (39.1) | 7 (10.9) | - |
| Other individual regimens | 8 | 4 (50.0) | - | 2 (25.0) | 1 (12.5) |

^§^Pre- and post-2010 indicate the periods of 2004–2010 and 2011–2018, respectively. ^¶^Percentages for each row are calculated using the total number of patients in the second column as the denominator; percentages do not add-up to 100.0% as not all second-line treatment options are presented. ASCT=autologous stem cell transplantation; Bor=bortezomib; Chemo=chemotherapy; Dara=daratumumab; IMiD= immunomodulatory drugs; N=number of patients.

**Table S16: Median follow-up, overall and by first-line treatment initiation period**

|  | **2004–2018** | **Pre-2010** | **Post-2010** |
| --- | --- | --- | --- |
| FU: Total number of patients (Events - Censored) | 4294 (2042–2252) | 1380 (405–975) | 2914 (1637–1277) |
| **Median FU in months (95% CI)** | **54.49 (51.41–57.84)** | **125.74 (122.66–129.84)** | **36.30 (33.93–38.00)** |
| 3 months FU rate in % (95% CI) | 96.79 (96.19–97.30) | 99.70 (99.20–99.89) | 95.38 (94.52–96.12) |
| 6 months FU rate in % (95% CI) | 93.37 (92.52–94.13) | 99.44 (98.82–99.73) | 90.40 (89.17–91.49) |
| 9 months FU rate in % (95% CI) | 89.96 (88.92–90.91) | 99.16 (98.44–99.55) | 85.42 (83.93–86.78) |
| 12 months FU rate in % (95% CI) | 86.87 (85.69–87.96) | 98.97 (98.18–99.41) | 80.89 (79.21–82.44) |
| 24 months FU rate in % (95% CI) | 75.85 (74.30–77.31) | 97.80 (96.70–98.54) | 64.89 (62.78–66.91) |
| 60 months FU rate in % (95% CI) | 46.39 (44.39–48.37) | 93.64 (91.73–95.12) | 22.77 (20.69–24.91) |

CI=Confidence interval; FU=Follow-up

**Table S17: Time-to-Event outcomes, overall and by first-line treatment initiation period**

|  | **2004–2018, N (%)** | **Pre-2010, N (%)** | **Post-2010, N (%)** |
| --- | --- | --- | --- |
| OS : Total number of patients (Events – Censored) | 4294 (2252–2042) | 1380 (975–405) | 2914 (1277–1637) |
| **Median OS in months (95% CI)** | **48.79 (45.15–51.67)** | **51.38 (47.28–57.70)** | **46.72 (41.25–52.20)** |
| 3 months OS rate in % (95% CI) | 85.71 (84.62–86.73) | 88.09 (86.26–89.69) | 84.55 (83.18–85.83) |
| 6 months OS rate in % (95% CI) | 78.72 (77.45–79.93) | 81.48 (79.32–83.43) | 77.38 (75.78–78.89) |
| 9 months OS rate in % (95% CI) | 74.38 (73.02–75.69) | 76.80 (74.48–78.95) | 73.21 (71.51–74.82) |
| 12 months OS rate in % (95% CI) | 71.51 (70.09–72.86) | 74.25 (71.85–76.47) | 70.15 (68.38–71.83) |
| 24 months OS rate in % (95% CI) | 62.59 (61.04–64.09) | 65.13 (62.54–67.59) | 61.31 (59.36–63.20) |
| 60 months OS rate in % (95% CI) | 44.44 (42.65–46.21) | 46.20 (43.51–48.84) | 43.44 (40.90–45.95) |
| ToT : Total number of patients (Events – Censored) | 3742 (3621–121) | 1232 (1232–0) | 2510 (2389–121) |
| **Median ToT in months (95% CI)** | **4.72 (4.36–4.95)** | **4.72 (4.23–4.95)** | **4.79 (4.30–4.95)** |
| 3 months ToT rate in % (95% CI) | 69.78 (68.27–71.23) | 70.13 (67.49–72.60) | 69.60 (67.75–71.38) |
| 6 months ToT rate in % (95% CI) | 30.35 (28.86–31.86) | 31.90 (29.31–34.51) | 29.54 (27.72–31.38) |
| 9 months ToT rate in % (95% CI) | 12.16 (11.11–13.25) | 19.32 (17.17–21.57) | 8.35 (7.28–9.52) |
| 12 months ToT rate in % (95% CI) | 8.27 (7.39–9.20) | 15.10 (13.16–17.16) | 4.61 (3.81–5.52) |
| 24 months ToT rate in % (95% CI) | 3.68 (3.09–4.33) | 6.90 (5.57–8.41) | 1.95 (1.44–2.58) |
| 60 months ToT rate in % (95% CI) | 0.86 (0.59–1.20) | 2.11 (1.42–3.03) | 0.18 (0.06–0.44) |

CI=Confidence Interval; N=Number of patients; OS=Overall survival; ToT=Time on Treatment

**Table S18 Early mortality rates overall and by first-line treatment initiation period**

|  |  | **2004–2018, N (%)** | **Pre-2010, N (%)** | **Post-2010, N (%)** |
| --- | --- | --- | --- | --- |
| Patients | N | 4,480 | 1,415 | 3,065 |
| Death prior to 3 months from first-line treatment initiation | Yes | 602 (13.4) | 161 (11.4) | 441 (14.4) |
|  | No | 3,701 (82.6) | 1,219 (86.1) | 2482 (81.0) |
|  | Clinical trial | 177 (4.0) | 35 (2.5) | 142 (4.6) |

N=number of patients.

**Table S19: Overall survival by first-line regimen group and by cardiac stage, 2004–2018**

| **Regimen group**^*^ | **IMiD-based** | **Bor-based** | **Chemo** |
| --- | --- | --- | --- |
| **All patients:** Total number of patients (Events - Censored) | 488 (346–142) | 2454 (1117–1337) | 869 (594–275) |
| **Median OS,** months (95% CI) | **48.56 (40.82–56.66)** | **45.28 (39.44–51.08)** | **41.05 (32.62–48.75)** |
|  |  |  |  |
| **Stage I: N**umber of patients (Events - Censored) **–** | 38 (19–19) | 355 (66–289) | 116 (53–63) |
| **Median OS** in months (95% CI) | **81.11 (51.08–NR)** | **NR (81.64–NR)** | **88.52 (66.98–NR)** |
|  |  |  |  |
| **Stage II**: number of patients (Events - Censored) | 88 (64–24) | 833 (298–535) | 271 (175–96) |
| **Median OS** in months (95% CI) | **39.41 (20.89–66.69)** | **66.95 (53.38–91.05)** | **44.75 (33.93–58.07)** |
|  |  |  |  |
| **Stage IIIa:** number of patients (Events - Censored) | **42 (33–9)** | **721 (375–346)** | **142 (106–36)** |
| **Median OS** in months (95% CI) | **7.34 (3.44–14.36)** | **32.75 (26.10–37.90)** | **14.33 (8.85–22.49)** |
|  |  |  |  |
| **Stage IIIb:** number of patients (Events - Censored) | 61 (54–7) | 435 (336–99) | 119 (99–20) |
| **Median OS** in months (95% CI) | **5.28 (2.39–13.05)** | **4.62 (3.48–5.67)** | **4.59 (2.56–6.00)** |

^*^Not all regimen groups are presented. Bor=bortezomib; Chemo=chemotherapy; CI=confidence interval; IMiD=immunomodulatory drug; OS=overall survival

**Table S20: Time-to-event outcomes for IgM-patients, overall and by first-line treatment initiation period**

|  | **2004–2018, N (%)** | **Pre-2010, N (%)** | **Post-2010, N (%)** |
| --- | --- | --- | --- |
| OS : Total number of patients (Events - Censored) | 193 (104–89) | 69 (46–23) | 124 (58–66) |
| **Median OS in months (95% CI)** | **39.74 (26.46–58.95)** | **51.70 (27.67–81.11)** | **29.77 (21.15–55.70)** |
| 3 months OS rate in % (95% CI) | 87.49 (81.91–91.43) | 85.51 (74.74–91.93) | 88.57 (81.46–93.07) |
| 6 months OS rate in % (95% CI) | 80.58 (74.20–85.53) | 79.68 (68.12–87.43) | 81.03 (72.83–86.97) |
| 9 months OS rate in % (95% CI) | 73.72 (66.74–79.46) | 75.26 (63.25–83.83) | 72.61 (63.48–79.82) |
| 12 months OS rate in % (95% CI) | 69.46 (62.17–75.61) | 70.83 (58.51–80.10) | 68.49 (58.98–76.24) |
| 24 months OS rate in % (95% CI) | 60.22 (52.44–67.14) | 66.40 (53.90–76.25) | 55.22 (44.77–64.48) |
| 60 months OS rate in % (95% CI) | 41.82 (33.58–49.83) | 46.87 (34.63–58.18) | 37.63 (26.03–49.18) |
| ToT : Total number of patients (Events - Censored) | 161 (157–4) | 61 (61–0) | 100 (96–4) |
| **Median ToT in months (95% CI)** | **4.03 (3.80–4.98)** | **4.03 (2.95–5.31)** | **4.07 (3.80–5.02)** |
| 3 months ToT rate in % (95% CI) | 67.45 (59.58–74.12) | 62.30 (48.92–73.10) | 70.64 (60.58–78.59) |
| 6 months ToT rate in % (95% CI) | 27.48 (20.77–34.60) | 31.15 (20.07–42.86) | 25.13 (16.99–34.09) |
| 9 months ToT rate in % (95% CI) | 14.06 (9.17–19.97) | 19.67 (10.85–30.41) | 10.47 (5.37–17.54) |
| 12 months ToT rate in % (95% CI) | 8.12 (4.49–13.11) | 11.48 (5.04–20.82) | 5.98 (2.34–12.11) |
| 24 months ToT rate in % (95% CI) | 1.35 (0.27–4.38) | 3.28 (0.61–10.09) | 0.00 (0.00–0.00) |
| 60 months ToT rate in % (95% CI) | 0.68 (0.06–3.40) | 1.64 (0.14–7.72) | 0.00 (0.00–0.00) |

CI=Confidence Interval; N=Number of patients; OS=Overall survival; PFS=Progression-Free survival; ToT=Time on Treatment

**Figures**

**Figure S1: Kaplan-Meier graph of overall survival (OS) by Mayo2012 revised cardiac stage at diagnosis**


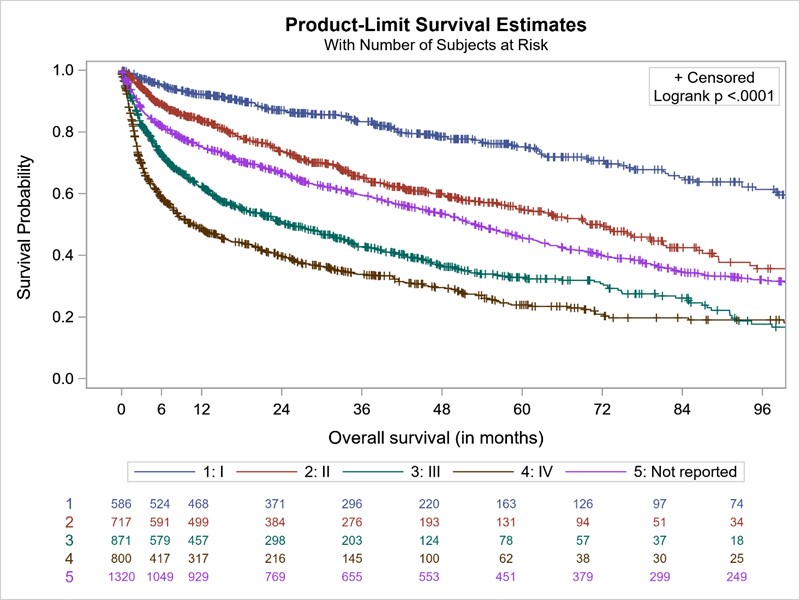


**Figure S2:** **Kaplan-Meier graph of OS by hematologic response at 6 months after first-line treatment initiation, 2004–2018**


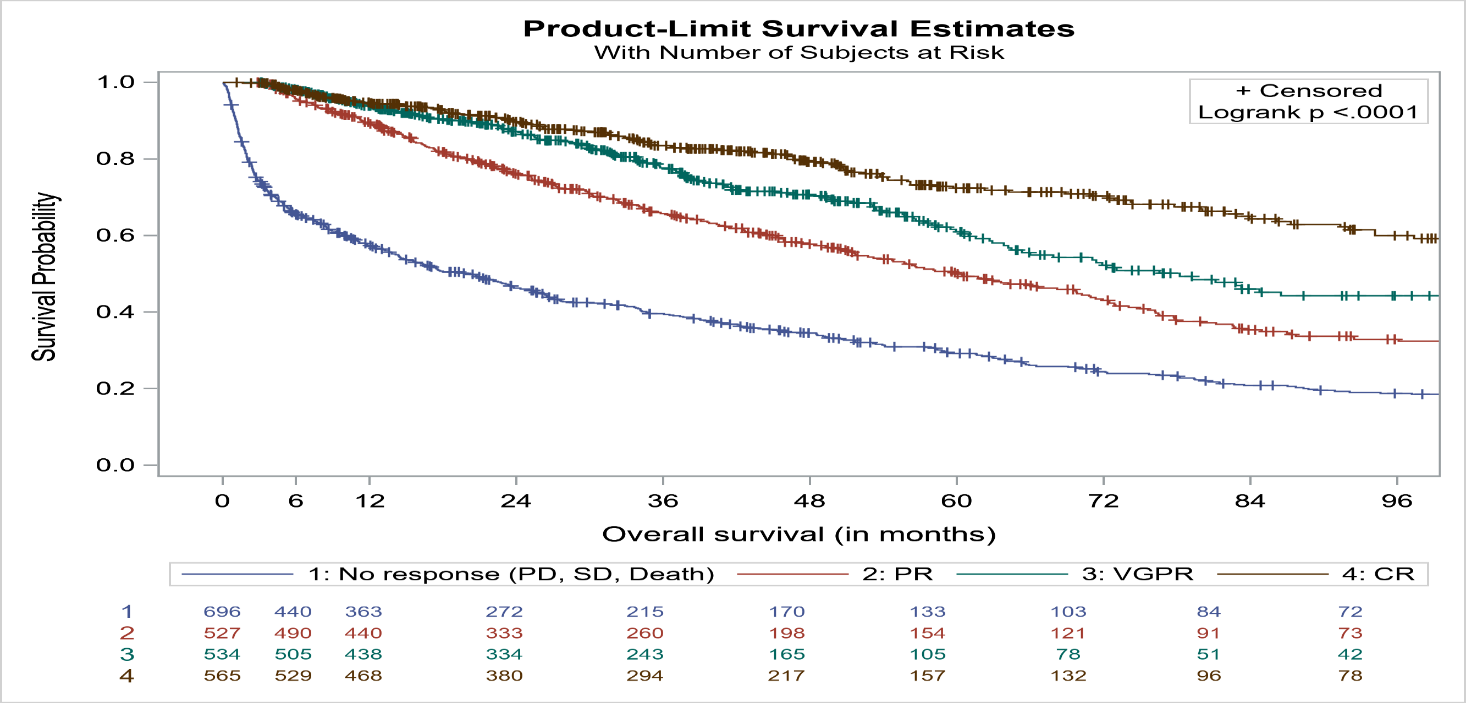


CR=complete response; PD=progressive disease; PFS=progression-free survival; PR=partial response; SD=stable disease; VGPR=very good partial response

**Figure S3: Kaplan-Meier graph of OS by hematologic response at 3 months after first-line treatment initiation, and by cardiac stage, 2004−2018**

**
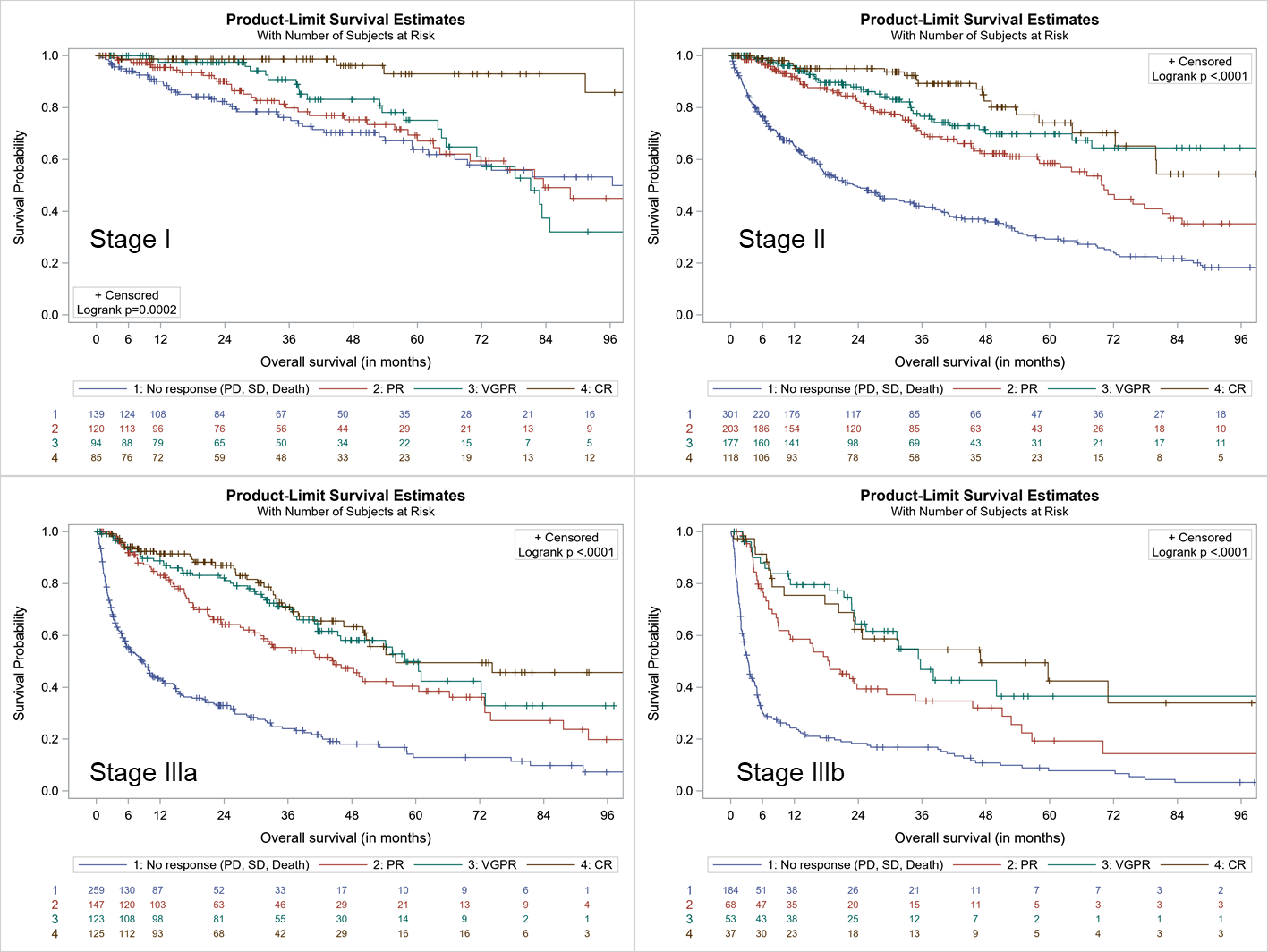
**

CR=complete response; PD=progressive disease; PFS=progression-free survival; PR=partial response; SD= stable disease; VGPR=very good partial response
